# Impact of two interventions on timeliness and data quality of an electronic disease surveillance system in a resource limited setting (Peru): a prospective evaluation

**DOI:** 10.1186/1472-6947-9-16

**Published:** 2009-03-10

**Authors:** Moises A Huaman, Roger V Araujo-Castillo, Giselle Soto, Joan M Neyra, Jose A Quispe, Miguel F Fernandez, Carmen C Mundaca, David L Blazes

**Affiliations:** 1 US Naval Medical Research Center Detachment, Lima, Peru; 2 Centro Medico Naval, Lima, Peru

## Abstract

**Background:**

A timely detection of outbreaks through surveillance is needed in order to prevent future pandemics. However, current surveillance systems may not be prepared to accomplish this goal, especially in resource limited settings. As data quality and timeliness are attributes that improve outbreak detection capacity, we assessed the effect of two interventions on such attributes in Alerta, an electronic disease surveillance system in the Peruvian Navy.

**Methods:**

40 Alerta reporting units (18 clinics and 22 ships) were included in a 12-week prospective evaluation project. After a short refresher course on the notification process, units were randomly assigned to either a phone, visit or control group. Phone group sites were called three hours before the biweekly reporting deadline if they had not sent their report. Visit group sites received supervision visits on weeks 4 & 8, but no phone calls. The control group sites were not contacted by phone or visited. Timeliness and data quality were assessed by calculating the percentage of reports sent on time and percentage of errors per total number of reports, respectively.

**Results:**

Timeliness improved in the phone group from 64.6% to 84% in clinics (+19.4 [95% CI, +10.3 to +28.6]; p < 0.001) and from 46.9% to 77.3% on ships (+30.4 [95% CI, +16.9 to +43.8]; p < 0.001). Visit and control groups did not show significant changes in timeliness. Error rates decreased in the visit group from 7.1% to 2% in clinics (-5.1 [95% CI, -8.7 to -1.4]; p = 0.007), but only from 7.3% to 6.7% on ships (-0.6 [95% CI, -2.4 to +1.1]; p = 0.445). Phone and control groups did not show significant improvement in data quality.

**Conclusion:**

Regular phone reminders significantly improved timeliness of reports in clinics and ships, whereas supervision visits led to improved data quality only among clinics. Further investigations are needed to establish the cost-effectiveness and optimal use of each of these strategies.

## Background

Surveillance systems provide essential data for the development and enhancement of disease prevention and control programs. They allow establishment of disease baselines, assessment of responses to public health measures and generation of hypotheses [1]. In addition, systems focused on emerging infectious disease surveillance are of special public health importance due to the potential for outbreaks that might lead to regional or even pandemic expansion if they are not rapidly controlled [2]. Thus, it is likely that containment of the next pandemic will primarily depend on current systems' capacity for timely and accurate detection of cases in all regions of the world. For example, estimations of pandemic influenza spread have shown that containment policies need to be established no more than 60 to 90 days after the occurrence of the first cases in order to avoid a country-wide expansion [3].

Even though national regulations often require medical providers to notify public health authorities of relevant infectious diseases, this process is incomplete and delayed even in developed countries where considerable amounts of resources are assigned to disease surveillance every year [4-6]. The situation is often more difficult in developing settings due to lack of trained personnel and lack of resources to ensure proper functioning of the system.

Many technological solutions have been developed to improve the performance of disease surveillance systems [7,8]. The implementation of electronic-based platforms, for instance, improves timeliness and facilitates access to epidemiological data allowing more rapid analysis and response [9-11]. In addition, enhancement of laboratory capacities and automated results reporting contributes to better data quality [12]. Comparisons between automated electronic laboratory reporting and traditional paper-based reporting have shown that automatizing the reporting process improves completeness of data and timeliness of reporting in disease surveillance [13,14]. However, new strategies must be further investigated, especially those aimed at reporting personnel, as they remain the most important component of disease surveillance systems, especially in resource-limited settings.

There are several attributes of surveillance systems that the CDC recommends be regularly assessed. Among these, data quality and timeliness are essential characteristics influenced by several factors that depend on reporting personnel [15,16]. For example, time constraints and confidentiality concerns worsen data quality and timeliness [17,18], whereas strengthening ties with reporting health care personnel improves notification rates by encouraging interactions between medical care and preventive health sectors [7,19].

Alerta is an electronic surveillance system for infectious diseases that has been successfully implemented in the Peruvian Navy and currently covers 97.5% of the Navy population [11]. Alerta was created in order to improve the detection, prevention and control of disease outbreaks in the Peruvian military settings. The Alerta system was conceived as a collaborative effort between the US Naval Medical Research Center Detachment (NMRCD) and the Peruvian Navy, with technological support of Voxiva. Since its creation in 2002, disease trends have been established for each of the 86 reporting units, allowing the detection of many outbreaks. Acute respiratory illnesses, diarrheal diseases and pneumonias are reported twice a week, whereas 38 other notifiable infectious diseases including malaria, dengue, leptospirosis, viral hepatitis, tuberculosis and others are reported immediately after their detection via phone, internet or radio relay. The data are then available in an internet-based platform with secure access in real-time.

Before health care personnel are assigned to reporting units, they are trained in epidemiological surveillance and the Alerta notification process. In order to maintain optimal notification in the Alerta system, regular monitoring of the reporting personnel is required; however, little data supporting the effectiveness of specific monitoring strategies is available in the literature. Given that telephone reminders and visits were used in the implementation phase of Alerta and these seemed to be useful in creating a surveillance culture among health care providers [11], we hypothesized that these interventions might be effective in improving timeliness and data quality among reporting units in the consolidation phase. Therefore, the objective of this study was to assess the impact of these two monitoring strategies aimed at the reporting personnel on timeliness and data quality of the Alerta system.

## Methods

We conducted a 12-week prospective study in 40 Alerta reporting units (18 clinics and 22 ships). Units enrolled were located in Lima-Callao (Peruvian capital) and had more than five months reporting to the Alerta system.

Reporting personnel responsible for surveillance in each unit participated in a 3.5-hour retraining course one week before the initiation of the study. The course program consisted of lectures about fundamentals of surveillance (1 hour) and case definitions of the most common notifiable diseases to Alerta (1 hour). In addition, hands-on sessions were conducted to practice how to notify via phone and internet (1.5 hours). Participants were provided with the Alerta system user's manual containing case definitions and description of the notification process. Reporting personnel were required to complete a self-administered questionnaire to assess factors related to deficient data quality and timeliness.

Units were then randomly assigned to one of three groups: phone, visit or control group. Reporting personnel in the phone group were contacted by telephone 3 hours before the biweekly deadline if they hadn't sent their reports at that point. A Peruvian Navy nurse from the Alerta Central Hub (ACH) was responsible for the monitoring process in all the units. He contacted the reporting personnel through their land-lines or mobile phones when appropriate. Phone conversations were based on an established template that included a reminder of the time deadline for the report and an opportunity for answering any questions: "Good Morning (title and last name). This is (navy nurse title and last name) from the Alerta Central Hub. You haven't sent the report of epidemiologic week (number) yet. Please, send your report today before 12:00 p.m. Do you have any questions/concerns regarding the notification? (Answer any questions). Thank you. Have a nice day". Reporting personnel in the visit group received a 30-minute supervision visit on week 4 and week 8 of intervention. The visit consisted of: (1) discussing the unit's notification performance during the last month; (2) retraining if needed in specific topics of the notification process; and (3) encouraging reporting personnel to report properly and on time. A team consisting of a physician and a navy nurse from the ACH performed the intervention visits. Units in the control group were not contacted by phone or visited during the study period. To avoid introduction of bias, reporting personnel were not told about the objective of the study or their group assignment. Phone calls and visits were considered part of the regular supervision activities of Alerta central hub personnel.

Timeliness was assessed through the report on time rate (ROTR), defined as the number of reports sent on time divided by the total number of reports, multiplied by 100. Data quality was assessed through errors per total number of reports (EPTR), defined as the number of errors detected in reports divided by the total number of reports, multiplied by 100. Trained personnel at the central hub reviewed the reports daily for data quality and error detection. Reporting personnel were contacted when reports seemed to contain erroneous data to confirm the accuracy of the information. Common errors included incorrect epidemiological weeks, duplication in reports, errors in case definition and erroneous number of cases.

Timeliness and data quality were assessed for acute respiratory illnesses, diarrheal diseases and pneumonias. Given that notification of these diseases was mandatory twice a week, ROTR and EPTR were also calculated twice per week, with a total of 24 measures of EPTR and ROTR in the 12-week pre-intervention period and another 24 measures of EPTR and ROTR during the 12-week intervention period for each reporting unit. Timeliness and data quality for other notifiable diseases such as malaria, dengue, viral hepatitis, tuberculosis and other infections that are reported immediately after their detection were not assessed given their expected very low incidence during the study period.

The data was entered into an MS-Excel 2000 database and analyzed in STATA 8.0. Data was described using central tendency measures with confidence intervals at 95%. The effect on EPTR and ROTR at each intervention group was modeled using generalized linear models (GLM) for binomial data (link function identity), clustered by site. GLMs were also used to compare the effect on EPTR and ROTR between groups. P-values < 0.05 were considered significant.

## Results

Baseline characteristics of the reporting units are shown in Table 1. No baseline differences were found between the intervention groups in terms of number of reporting personnel, reporting tools available and time operating Alerta. However, when we compared these characteristics according to the type of unit (clinics and ships), there were differences in the number of reporting personnel (p = 0.007), reporting tools available (p = 0.052) and time operating the Alerta system (p = 0.005).

**Table 1:** Baseline characteristics of reporting units

Baseline characteristics	All	p-value (*)	Phone	Visits	Control	p-value (**)
**Clinics **(n = 18)						
Number of reporting personnel (median)	4	0.007	3	4	3	0.799
Reporting media (# Units)		0.052				0.435
Phone	6		3	1	2	
Internet	3		1	2	0	
Phone & Internet	9		2	3	4	
Time in Alerta (months, median)	21	0.005	28.5	21.5	22	0.703
**Ships **(n = 22)						
Number of reporting personnel(median)	2		3	2	2	0.922
Reporting media (# Units)						0.854
Phone	14		5	5	4	
Internet	0		0	0	0	
Phone & Internet	8		3	2	3	
Time in Alerta (months, median)	19		20	19	18	0.994

(*) p-value obtained when comparing clinics and ships

(**) p-value obtained when comparing phone, visits and control group in each type of unit (clinic or ship).

All reporting personnel responsible for coordinating surveillance in the selected units participated in the short retraining course and completed the questionnaire about factors related to deficient data quality and timeliness (Table 2). Absence due to military missions or assignments, limited access to reporting tools and forgetfulness in reporting were the most frequent factors related to delayed reports. Lack of training and generation of errors by the system itself were the most important factors related to reporting errors, according to the personnel.

**Table 2:** Survey results: Factors related to delayed reports and errors in reports

Related Factors	% Among Responders n = 44 % (C.I.)
**Delayed reports**	
Away due to Military assignments	68.2 (52.4 – 81.4)
Limited access to communication tools (phone, PC)	40.9 (26.3 – 56.8)
Forgetfulness in reporting	36.4 (22.4 – 52.2)
Electronic system's deficiencies	25.0 (13.2 – 40%)
Time constraints	18.2 (8.2 – 32.7)
Lack of training	2.3 (0.1 – 12)
**Errors in reports**	
Lack of training	36.4 (26.4 – 47.3)
Electronic system's deficiencies	30.7 (21.3 – 41.4)
Time constraints	26.1 (17.3 – 36.6)
Limited access to notification guidelines	18.2 (10.8 – 27.8)

When we compared the pre-intervention and during-intervention timeliness of reports in each group, the phone group showed a significant increase in ROTR (p < 0.001). This improvement was also maintained after stratifying groups according to type of unit (clinic or ship) (Table 3). Neither the visit group nor the control group showed a significant improvement in timeliness (p = 0.798; p = 0.847 respectively). Generalized linear models were used to compare the effects in ROTR between the intervention groups. To demonstrate that different effects were not due to the differences in baseline ROTR levels, an interaction between time (pre-intervention & during intervention) and group (phone, visit and control) was introduced into the model. The analysis showed that the interaction between time and phone group had a significant effect in ROTR among clinics (β = 0.2153; p = 0.005) and ships (β = 0.3067; p = 0.002).

**Table 3:** Effect of phone calls and supervision visits on timeliness, stratified by type of site

		Report on Time Rate* (ROTR)		Variation ROTR	
				
Type Site	Group	Pre Intervention %	Intervention %	Δ (CI)	p-value
				
Clinic	Phone	64.6	84.0	↑19.4 (↑10.3 – ↑28.6)	<0.001
	Visits	60.4	60.4	0 (↓22.3 -↑22.3)	1.000
	Control	59.0	56.9	↓2.1 (↓18.9 - ↑14.7)	0.808
Ship	Phone	46.9	77.3	↑30.4 (↑16.9 – ↑43.8)	<0.001
	Visits	54.2	57.1	↑2.9 (↓11.3 – ↑17.2)	0.0682
	Control	53.0	52.7	↓0.25 (↓15.6 - ↑15.1)	0.975

(*) Report on time rate (ROTR) = Number of reports sent on time divided by the total number of reports, multiplied by 100.

To explore in detail the effect on timeliness after each visit in the visit group, a temporal analysis was performed looking for any gradual changes in ROTR between periods 0-to-4 week, 5-to-8 week and 9-to-12 week. No significant differences were found between these periods (p = 0.673; p = 0.632).

Regarding the data quality assessment, the visit group showed a decrease in the EPTR (p = 0.017). However, after stratifying groups by type of site, the decrease in EPTR persisted in clinics (p = 0.007), but was not evident in ships (p = 0.455) (Table 4). Neither the phone group nor control group showed a significant decrease in error rates (p = 0.241, p = 0.309 respectively). Again, using generalized linear models, we introduced an interaction between time and group to evaluate the effect in EPTR. The analysis showed that the interaction between time and visit group had a significant effect in EPTR among clinics (β = -0.0418; p = 0.049), but not among ships (β = 0.0041; p = 0.820).

**Table 4:** Effect of phone calls and supervision visits on data quality, stratified by type of site

		Error per Total Reports* (EPTR)		Variation EPTR	
				
Type Site	Group	Pre Intervention %	Intervention %	Δ**(CI)**	p-value
				
Clinic	Phone	0.99	1.06	↑0.07 (↓0.9 – ↑1.0)	0.888
	Visits	7.10	2.00	↓5.10 (↓8.7 - ↓1.4)	0.007
	Control	1.94	1.02	↓0.92 (↓3.4 - ↑1.5)	0.470
Ship	Phone	8.16	6.69	↓1.47 (↓4.6 - ↑1.7)	0.361
	Visits	7.32	6.67	↓0.65 (↓2.4 - ↑1.1)	0.455
	Control	8.49	7.59	↓0.90 (↓3.6 - ↑1.8)	0.519

(*) Error per total reports (EPTR) = Number of errors detected in reports divided by the total number of reports, multiplied by 100

## Discussion

This study showed that regular phone call reminders significantly enhanced timeliness of reports in this particular electronic disease surveillance system. Our findings suggest that phone calls might correct forgetfulness of notification, which was identified as an important determinant of reporting delays by surveillance personnel. Given that health care providers in clinical settings are usually overloaded with many tasks, disease reporting is not considered a top priority, particularly in developing countries. Thus, contacting reporting personnel a few hours before the deadline was shown to be the key strategy to reach better report on time rates, even when the duration of phone calls was less than two minutes on average. On the other hand, monthly supervision visits allowed closer contact with reporting personnel, but did not show significant improvement in timeliness, probably because of their limited frequency. Occasional visits to those who provide the data is a common activity in successfully implemented systems [2]; however, its use should be carefully considered by system administrators when the priority is improvement in timeliness.

There are factors intrinsically related to military settings that might have contributed negatively to timely surveillance in this sample. For example, frequent deployments away from home and restriction of communications during missions could generate unintentional epidemiological silence. In addition, limited access to reporting tools such as computers with internet access also affects timeliness in electronic-based surveillance systems, especially in developing countries where availability of technology is not widespread [20].

Despite the fact that no outbreaks were detected during the study, it logically follows that enhancing timeliness in the phone group would have led to more rapid outbreak detection. Surveillance systems that diminish notification delays improve their capacity for detecting outbreaks and allow the triggering of more appropriate responses [9,16,21]. Military and other confined populations are of special concern because they are often exposed to conditions favoring the transmission and perpetuation of pathogens [22]. Moreover, the need for early disease detection systems in these settings is increasing as the potential for pandemics persists and studies have shown that military populations played an important role in the origin and explosive expansion of pandemic agents in the past – in the US, the influenza pandemic of 1918–19 began among military trainees [23,24].

The effect of supervision visits on data quality differed in clinics when compared to ships. Previous studies have shown that regular training decreases reporting errors [25,26]; however, in our study, there was no beneficial effect of briefly training personnel during supervision visits performed on the ships. It is possible that because ships had less experience (time) using the Alerta system, visits did not improve data quality on the ships as they did in the clinics. More intensive monitoring and training may be needed in these newly incorporated sites. The lack of improvement in data quality on the ships might also be partially explained by inherent adverse conditions that exist on Navy ships, such as frequent personnel turnover, frequent accidents and frequent deployments with limited access to assistance.

Although electronic-based notification often enhances data quality [9-11], Alerta reporting personnel perceived that the system's platform itself generated errors. However, the data did not corroborate this perception. Perceived barriers for the implementation of biotechnologies have been described in developing countries [20], including the difficulty in replacing traditional pen and paper reporting systems with technological solutions that are not commonly used. This negative perception of technology is very important to address in order to assure successful adoption of technology among users and thus favor the optimal implementation of an electronic-based system in developing countries [27].

This study had some limitations. We performed 170 calls in the phone group during the study period, but 16.5% of the calls were not successful (reporting personnel did not answer or were absent from the unit despite at least three attempts to contact them before time deadline). Although there was a small sample size, we did a total of 24 measures of EPTR and ROTR before and during the interventions for each site. The use of generalized linear models (GLM) for binomial data allowed us to calculate the impact of the interventions considering these pre and post measures in each reporting unit maintaining a clustered analysis. Finally, specific characteristics in this sample might affect the reproducibility of our results in other settings. For example, health care providers in the Alerta system are also charged with disease notification, which differs from the majority of other surveillance systems in the region where administrative personnel provide notification. In addition, constant deployments and frequent missions might negatively affect timeliness and data quality of reports in this sample, especially among ships when they are underway.

Further investigations are needed to establish the cost-effectiveness and optimal use of telephone reminders and visits. Moreover, interventions such as automated telephone or text messaging reminders and retraining/monitoring through video-teleconference might be explored. However, potential perception barriers against these technological solutions should be considered in resource-limited settings when trying to apply automated interventions. With respect to this, we feel that the person-to-person interaction during the telephone reminders and visits played an important role in our results, as they allowed strengthening ties between reporting personnel and the central hub and possibly enhanced the perception of ownership among reporting personnel.

## Conclusion

This study demonstrates that regular phone reminders significantly improved the reporting timeliness in clinics and ships, whereas monitoring visits improved data quality only in clinics in the Alerta system. Further investigations are needed to establish the cost-effectiveness and optimal use of each of these strategies. Applying automated text messaging reminders and providing retraining/monitoring through video-teleconference are possible next steps.

## Competing interests

The authors declare that they have no competing interests.

## Authors' contributions

MAH conceived of the study, supervised all aspects of its conduction and wrote the manuscript. DLB and RVA assisted with the methodology, synthesized analysis and led to writing. GS and CCM assisted with the analysis and conduction of the study. JMN and JAQ assisted with the conduction of the study. MFF provided the facilities to the development of the system. All authors helped to conceptualize ideas, interpret findings and review drafts of the manuscript. All authors read and approved the final manuscript.

## Pre-publication history

The pre-publication history for this paper can be accessed here:

http://www.biomedcentral.com/1472-6947/9/16/prepub
